# The *MAT* Locus Genes Play Different Roles in Sexual Reproduction and Pathogenesis in *Fusarium graminearum*

**DOI:** 10.1371/journal.pone.0066980

**Published:** 2013-06-24

**Authors:** Qian Zheng, Rui Hou, Juanyu, Zhang, Jiwen Ma, Zhongshou Wu, Guanghui Wang, Chenfang Wang, Jin-Rong Xu

**Affiliations:** 1 State Key Laboratory of Crop Stress Biology for Arid Areas, College of Plant Protection, Northwest Agricultural and Forestry University, Yangling, Shaanxi, China; 2 Department of Botany and Plant Pathology, Purdue University, West Lafayette, Indiana, United States of America; University of Wisconsin - Madison, United States of America

## Abstract

Sexual reproduction plays a critical role in the infection cycle of *Fusarium graminearum* because ascospores are the primary inoculum. As a homothallic ascomycete, *F. graminearum* contains both the *MAT1-1* and *MAT1-2-1* loci in the genome. To better understand their functions and regulations in sexual reproduction and pathogenesis, in this study we assayed the expression, interactions, and mutant phenotypes of individual *MAT* locus genes. Whereas the expression of *MAT1-1-1* and *MAT12-1* rapidly increased after perithecial induction and began to decline after 1 day post-perithecial induction (dpi), the expression of *MAT1-1-2* and *MAT1-1-3* peaked at 4 dpi. *MAT1-1-2* and *MAT1-1-3* had a similar expression profile and likely are controlled by a bidirectional promoter. Although none of the *MAT* locus genes were essential for perithecium formation, all of them were required for ascosporogenesis in self-crosses. In outcrosses, the *mat11-1-2* and *mat11-1-3* mutants were fertile but the *mat1-1-1* and *mat1-2-1* mutants displayed male- and female-specific defects, respectively. The *mat1-2-1* mutant was reduced in *FgSO* expression and hyphal fusion. Mat1-1-2 interacted with all other *MAT* locus transcription factors, suggesting that they may form a protein complex during sexual reproduction. Mat1-1-1 also interacted with FgMcm1, which may play a role in controlling cell identity and sexual development. Interestingly, the *mat1-1-1* and *mat1-2-1* mutants were reduced in virulence in corn stalk rot assays although none of the *MAT* locus genes was important for wheat infection. The *MAT1-1-1* and *MAT1-2-1* genes may play a host-specific role in colonization of corn stalks.

## Introduction

*Fusarium graminearum* (teleomorph *Gibberella zeae*) is a causal agent of Fusarium head blight (FHB) or scab of wheat, barley, and other small grains worldwide [1], [2]. This fungal pathogen overwinters on plant debris and produces ascospores in the spring to initiate infection of flowering tissues of wheat and other host plants. Unlike most pathogenic fungi, sexual reproduction plays a critical role in the infection cycle of *F. graminearum* because ascospores are the primary inoculum [3]. Flowering wheat heads are susceptible to Fusarium infection from anthesis through the milk stages. Under favorable environmental conditions, FHB can cause severe yield losses. In addition, *F. graminearum* is a producer of deoxynivalenol (DON) and zearalenone. Infested grains are often contaminated with these harmful mycotoxins [4].

As a homothallic ascomycete, *F. graminearum* has both *MAT1-1* and *MAT1-2-1* that are arranged in tandem [5]. Similar to mutants deleted of the entire mating locus, the *mat1-1* and *mat1-2-1* deletion mutants were sterile in self-crosses [6]. Similar to many other Sordariomycetes, *MAT1-1* of *F. graminearum* contains three putative transcription factor (TF) genes known as *MAT1-1-1*, *MAT1-1-2*, and *MAT1-1-3* [5]. Mat1-1-1 is the well-conserved alpha1 domain protein but the putative DNA-binding domain of Mat1-1-2 or Mat1-1-3 (HMG-like) has not been functionally characterized. In contrast, *MAT1-2-1* contains a single transcription factor gene that encodes a HMG-box protein. Recently, it has been shown that alpha domain proteins and HMG proteins encoded by fungal mating type genes are evolutionally related [7]. The *MAT1-2-3* gene adjacent to *MAT1-2-1* was predicted to be a new *MAT* gene [8]. However, unlike other *MAT* TF genes, *MAT1-2-3* has no DNA-binding motif. Deletion of *MAT1-2-3* had no effects on sexual reproduction [9].

The functions of *MAT* locus genes have been characterized in several Sordariomycetes, including *Neurospora crassa, Podospora anserina,* and *Sordaria macrospora* [10], [11]. In general, the *MAT* TFs are dispensable for vegetative growth and asexual reproduction. In most filamentous ascomycetes, the *MAT1-1-1* and *MAT1-2-1* orthologs are essential for mating and mating type specificity. In *N. crassa*, the *matA-1* and *mata-1* mutants were sterile but the *matA-2* and *matA-3* mutants were only slightly reduced in fertility [12]. Even the *matA-2 matA-3* double mutant was still fertile although it was significantly reduced in perithecium and ascospore formation, suggesting that these genes may have overlapping functions in sexual development. In *S. macrospora*, a homothallic ascomycete closely related to *N. crassa*, the *SmtA-1* (*MAT1-1-1*) and *SmtA-3* (*MAT1-1-3*) deletion mutants produced the same number of perithecia and mature, viable ascospores as the wild type. In contrast, mature perithecia were not observed in the *SmtA-2* (*MAT1-1-2*) deletion mutant, which was blocked in sexual development at the stage of early protoperithecium formation [10].

In *Saccharomyces cerevisiae*, only the *MAT* locus genes in the *MAT* locus are transcribed. The *MAT* genes in the *HML* and *HMR* loci are silenced [13], [14]. The silenced *HMLα* or *HMLa* loci function as the donor sequences for gene conversion during mating type switching. In *MATα* cells, Matα1 interacts with Mcm1 to express α-specific genes and MATα2 inhibits the *a*-specific genes by binding with Mcm1/Tup1/Ssn6. In diploid cells, Mata1 interacts with Matα2 to inhibit haploid-specific genes, such as *a*- or *α-*specific pheromone and pheromone receptor genes [15], [16], [17]. Mating type switching has not been observed in filamentous fungi although some homothallic fungi contain both *MAT* idiomorphs in the genome. In *N. crassa*, the interaction between *mat*A-1 and *mat*a-1 proteins was detected [18]. The *mat*A-2 and *mat*A-3 proteins weakly interact with each other in yeast two-hybrid assays [19]. In *S. macrospora*, SmtA-1 is known to interact with Mcm1 and Smta-1 but not with SmtA-2 or SmtA-3 [20], [21]. In the heterothallic ascomycete *P. anserina*, no direct interactions among *MAT* locus TF proteins encoded by the opposite mating type loci were detected in yeast two-hybrid assays. Nevertheless, the *mat-A* specific transcription factors *FMR1* and *SMR2* that are orthologous to *matA-1* and *matA-3* of *N. crassa* interacted with each other [22].

The *MAT* locus genes are well conserved in *F. graminearum*. Because proper regulation of the expression, activation, and interaction of *MAT* locus genes is important for growth and differentiation in a homothallic fungus, in this study we functionally characterized individual *MAT* locus genes. All four *MAT* locus genes in *F. graminearum* had induced expression during sexual reproduction and were required for ascosporogenesis. However, none of them was essential for growth and infection of flowering wheat heads, which is similar to a recent report on functional characterization of *MAT* locus genes [9] that was published during the preparation of this manuscript. Although we failed to detect the direct interaction between Mat1-1-1 and Mat1-2, Mat1-1-2 interacts with all other *MAT* locus genes in yeast two hybrid-assays, suggesting that they may form protein complexes during sexual reproduction. Both *MAT1-1-1* and *MAT1-2-1* were rapidly induced during early stages of sexual development then gradually declined. The expression of *MAT1-1-2* and *MAT1-1-3* peaked at 4 days post-perithecial induction, which was later than the up-regulation of *MAT1-1-1* and *MAT1-2-1*. *MAT1-1-2* and *MAT1-1-3* had similar expression profiles and they may have overlapping functions during late stages of perithecium development. In addition to the male- and female-specific defects observed in the *mat1-1-1* and *mat1-2-1* deletion mutants, respectively, we found that *MAT1-1-1* and *MAT1-2-1* are important for colonization of corn stalks, suggesting that they play a host-specific role in pathogenesis.

## Results

### Differential Expression of*MAT* Locus Genes in *F. graminearum*

**Figure 1:**
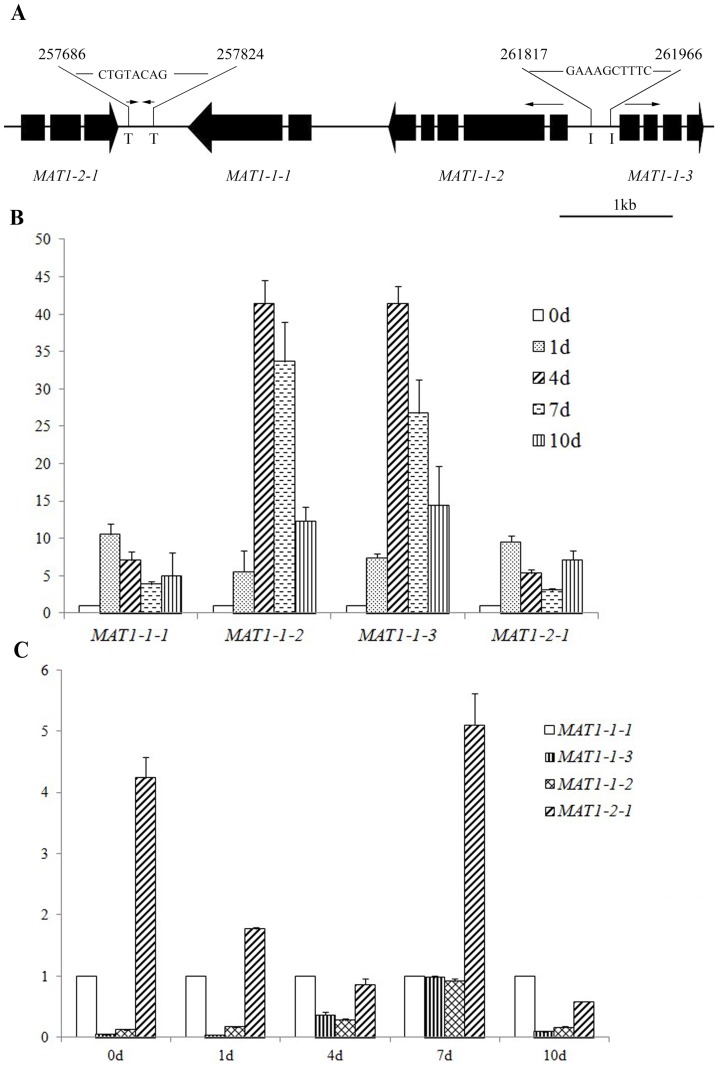
Chromosomal positions and expression levels of*MAT* locus genes. **A.** **** Diagram of four *MAT* locus genes. The thick black bars and arrows represent the exons and directions of *MAT1-2-1, MAT1-1-1, MAT1-1-2,* and *MAT1-1-3* genes. I and T represent the initiation and termination sites of transcription. CTGTACAG and GAAAGCTTTC are palindromic sequences. **B**. Relative expression levels of individual *MAT* locus genes at 1, 2, 4, 6, and 10 days post-perithecial induction (dpi) were assayed by qRT-PCR. The expression level of individual genes in aerial hyphae collected from 7-day-old carrot agar cultures before perithecial induction (day 0 control) was arbitrarily set to 1. **C**. Relative expression levels of four MAT locus TF genes at 1, 2, 4, 6, and 10 dpi. For each time point, the expression level of *MAT1-1-1* was set to 1. Mean and standard error were calculated from three independent biological replicates.

In the closely clinked *MAT* idiomorphs, the *MAT1-2-1* (FGSG_008993) and *MAT1-1-1* (FGSG_008992) genes share a 139-bp terminator region that contains a CTGTACAG palindromic sequence. In contrast, *MAT1-1-2* (FGSG_008991; 259983-261563–) and *MAT1-1-3* (FGSG_008990; 262020-262759+) have the head-to-head arrangement and are only 457-bp apart (Fig. 1A), suggesting that they share a bidirectional promoter [23]. In comparison with the corresponding ESTs, the putative transcription initiation sites of these two genes are at 261816 and 262075, respectively. The 259-bp sequence between the transcription initiation sites of *MAT1-1-2* and *MAT1-1-3* contains a palindromic GAAAGCTTTC sequence, which consists of two CTTTC sequences in different strands. In *N. crassa*, the *mata*-1 binding site is CTTTG [24].

To determine the expression profiles of *MAT* locus genes during sexual reproduction, we isolated RNA from aerial hyphae scrapped off 7-day-old carrot agar plates and fungal biomasses harvested from carrot agar cultures 1, 2, 4, 6, and 10 days post-perithecial induction (dpi). In aerial hyphae harvested before perithecial induction, the expression of *MAT* locus genes was not detectable or relatively low (Fig. 1B), suggesting that their expression was suppressed during vegetative growth. The expression of *MAT1-1-1* and *MAT1-2-1* increased approximately 11- and 10-fold, respectively at 1 dpi, and decreased after that (Fig. 1B). Therefore, these two *MAT* locus TF genes are likely important for earlier stages of sexual reproduction. For the *MAT1-1-2* and *MAT1-1-3* genes, their expression also increased after perithecial induction but peaked at 4 dpi (Fig. 1B). A similar expression profile of *MAT1-1-2* and *MAT1-1-3* is consistent with the fact that they share a common promoter (Fig. 1). These two genes may play more important roles in later stages than in earlier stages of perithecium development. When compared with the expression levels of other *MAT* TF genes, *MAT1-2-1* had the highest level at 0, 1, 4, and 7 dpi (Fig. 1C). *MAT1-1-1* had a higher expression level than *MAT1-1-2* and MAT1-1-3 at 0, 1, 4, and 10 dpi (Fig. 1C). Similar trends were observed in an earlier study [9].

### Expression and Subcellular Localization of*MAT* Locus Genes

**Figure 2:**
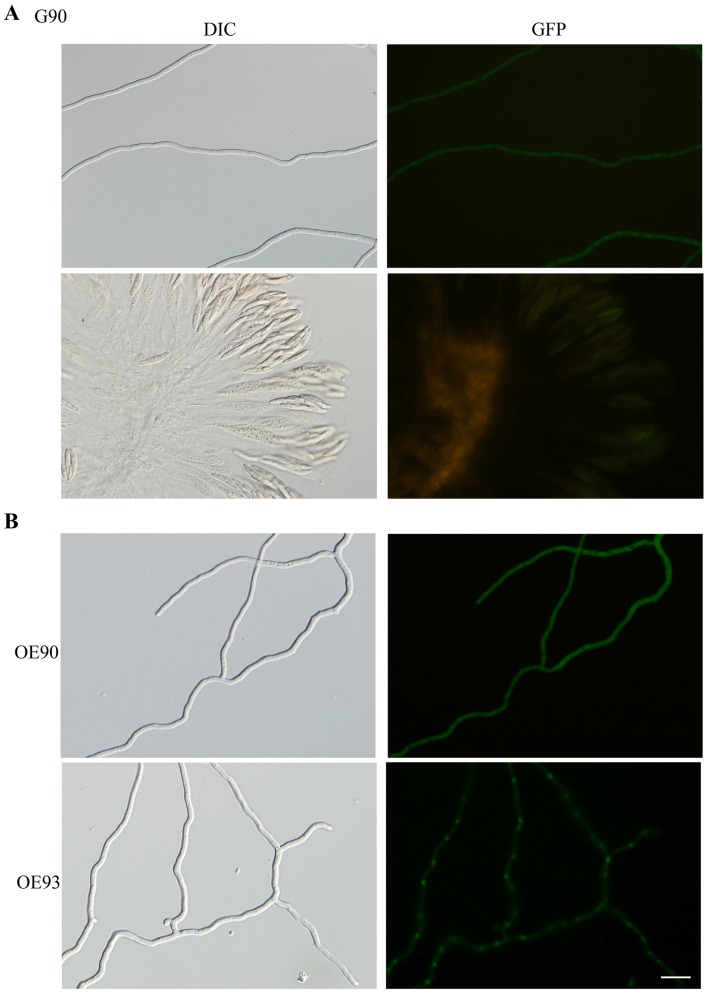
Expression and subcellular localization of*MAT* locus TF genes. **A** ****. Weak GFP signals in vegetative hyphae (upper panels) and asci (lower panels) of the *MAT1-1-3*-GFP knock-in transformant. **B**. GFP signals in vegetative hyphae of the P_TrpC_-*MAT1-1-3-*GFP (90R) and P_TrpC_-*MAT1-2-1*-GFP (93R) transformants. The same field was observed by DIC or epifluorescence microscopy. Bar = 20 µm.

To determine their expression and subcellular localization during sexual development, we generated the knock-in GFP fusion transformants for the four *MAT* locus TF genes of *F. graminearum* (Fig. S1). All the resulting GFP knock-in transformants (Table 1) were normal in vegetative growth and sexual reproduction, indicating that GFP insertion had no obvious effects on their functions. However, GFP signals were very weak (Fig. 2A) or not detectable (Fig. S1) in vegetative hyphae or ascogenous tissues in all these knock-in transformants, including the *MAT1-1-3-*GFP strain (Fig. 2A). Weak GFP signals were too faint to be distinguished from the auto-fluorescence of perithecium tissues and we failed to detect the localization of GFP signals in the nucleus in any of these GFP knock-in transformants.

**Table 1:** Strains used in this study.

Strains	Genotype	References
PH-1	Wild type	[53]
*mat1-1*	*mat1-1* deletion mutant of PH-1	[6]
M92-2	*mat1-1-1* deletion mutant of PH-1	This study
M92-5	*mat1-1-1* deletion mutant of PH-1	This study
M92-15	*mat1-1-1* deletion mutant of PH-1	This study
M91-3	*mat1-1-2* deletion mutant of PH-1	This study
M91-8	*mat1-1-2* deletion mutant of PH-1	This study
M91-21	*mat1-1-2* deletion mutant of PH-1	This study
M90-1	*mat1-1-3* deletion mutant of PH-1	This study
M90-11	*mat1-1-3* deletion mutant of PH-1	This study
M90-12	*mat1-1-3* deletion mutant of PH-1	This study
M90-20	*mat1-1-3* deletion mutant of PH-1	This study
M93-1	*mat1-2-1* deletion mutant of PH-1	This study
M93-15	*mat1-2-1* deletion mutant of PH-1	This study
M93-7	*mat1-2-1* deletion mutant of PH-1	This study
S6	*Fgso* deletion mutant of PH-1	This study
G92-9	*MAT1-1-1-*GFP knock-in transformant	This study
G91-11	*MAT1-1-2-*GFP knock-in transformant	This study
G91-21	*MAT1-1-2-*GFP knock-in transformant	This study
G90	*MAT1-1-3-*GFP knock-in transformant	This study
G93	*MAT1-2-1-*GFP knock-in transformant	This study
B7-1	*MAT1-1-1-*YFPN+*MAT1-1-2*-YFPC transformant	This study
B7-2	*MAT1-1-1*-YFPN+*MAT1-1-2*-YFPC transformant	This study
B8-1	*MAT1-2-1-*YFPN+*MAT1-1-2*-YFPC transformant	This study
B8-2	*MAT1-2-1-*YFPN+*MAT1-1-2*-YFPC transformant	This study
B9-1	*MAT1-1-3*-YFPN+*MAT1-1-2*-YFPC transformant	This study
B9-2	*MAT1-1-3*-YFPN+*MAT1-1-2*-YFPCtransformant	This study
B10-1	*MAT1-1-3*-YFPC+*MAT1-2-1-*YFPC transformant	This study
B10-2	*MAT1-1-3-*YFPN+*MAT1-2-1-*YFPCtransformant	This study
OE90	P_TrpC_-*MAT1-1-*3-GFP transformant of PH-1	This study
OE93	P_TrpC_-*MAT1-2-1*-GFP transformant of PH-1	This study
Comp90	*mat1-1-3/MAT1-1-3*-GFP complemented transformant	This study
Comp91	*mat1-1-2/MAT1-1-2-*GFP complemented transformant	This study

In addition to the knock-in transformants, we also generated transformants expressing the P_TrpC_-*MAT1-1-3*-GFP and P_TrpC_-*MAT1-2-1*-GFP fusion constructs ectopically. In the P_TrpC_-*MAT1-1-3*-GFP transformant, GFP signals in the cytoplasm were still relatively weak but appeared to be stronger than the knock-in transformant (Fig. 2B). Localization of GFP-signals to the nucleus was observed in vegetative hyphae of the *MAT1-2-1*-GFP transformant (Fig. 2B). Nevertheless, we still failed to observe GFP signals that were stronger than the fluorescent background in perithecium tissues of the P_TrpC_-*MAT1-1-3*-GFP and P_TrpC_-*MAT1-2-1*-GFP transformants. It is possible that the GFP fusion proteins of these *MAT* locus gene are not stable or degraded rapidly during sexual reproduction.

### Mutants Deleted of Individual*MAT* Locus Genes are Defective in Sexual Reproduction

**Figure 3:**
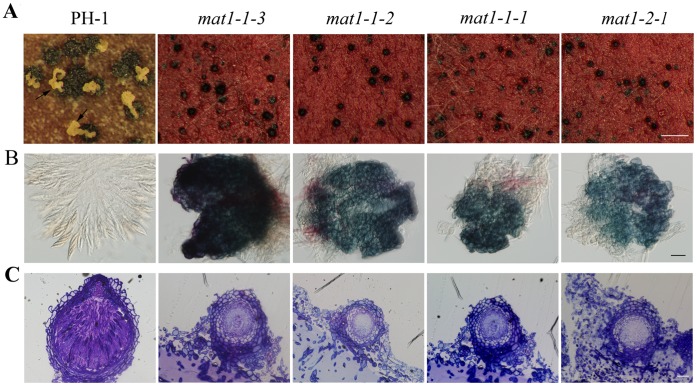
Defects of different*MAT* locus gene deletion mutants in self-crosses. **A** ****. Perithecia produced by 14-day-old carrot agar cultures of the wild type (PH-1) and different *MAT* locus gene deletion mutants. Arrows pointed to the cirrhi. Bar = 1 mm. **B**. Perithecia of PH-1 and the *MAT* locus gene deletion mutants were examined for ascus and ascospore development. Bar = 20 µm. **C.** Thick sections of representative perithecia produced by the wild type and mutant strains. Bar = 20 µm.

To determine the functions of *MAT* TFs, we generated gene replacement mutants deleted of individual *MAT* locus genes in the wild-type strain PH-1 (Fig. S2). For each gene, at least three independent knockout mutants were isolated (Fig. S2; Table 1) and found to have the same phenotypes described below (data not shown). None of the resulting mutants (Table 1) had obvious defects in the growth rate or colony morphology (Fig. S3). Conidiation and conidium morphology also were normal in these mutants (Fig. S4). Nevertheless, all the *MAT* locus gene deletion mutants were defective in sexual reproduction in self-crosses. At 7 days post-perithecial induction, all the mutants produced dark-pigmented perithecia that were smaller than the wild-type perithecia (Fig. 3A). These mutant perithecia were sterile and failed to produce ascospore cirrhi. When cracked open, asci or ascospores were not observed in perithecia formed by the mutants (Fig. 3B).

To further characterize the defects of *MAT* locus TF deletion mutants in sexual reproduction, thick-sections of the wild-type and mutant perithecia were examined under a light microscope (Fig. 3C). In contrast to ascogenous hyphae and asci at the base of the wild-type perithecia, perithecia formed by the *MAT* locus gene deletion mutants appeared to be blocked in the development of ascogenous hyphae and tended to have thicker layers of perithecium wall (Fig. 3C). No asci or ascospores were observed. These results indicate that none of the *MAT* locus genes is essential for the initiation of perithecium development. However, the development of asci or ascogenous hyphae and enlargement of young perithecia require proper functions of all four *MAT* locus genes in self-crosses.

### The*mat1-1-1* and *mat1-2-1* Deletion Mutants are Defective in Infection of Corn Stalks

**Figure 4.The:**
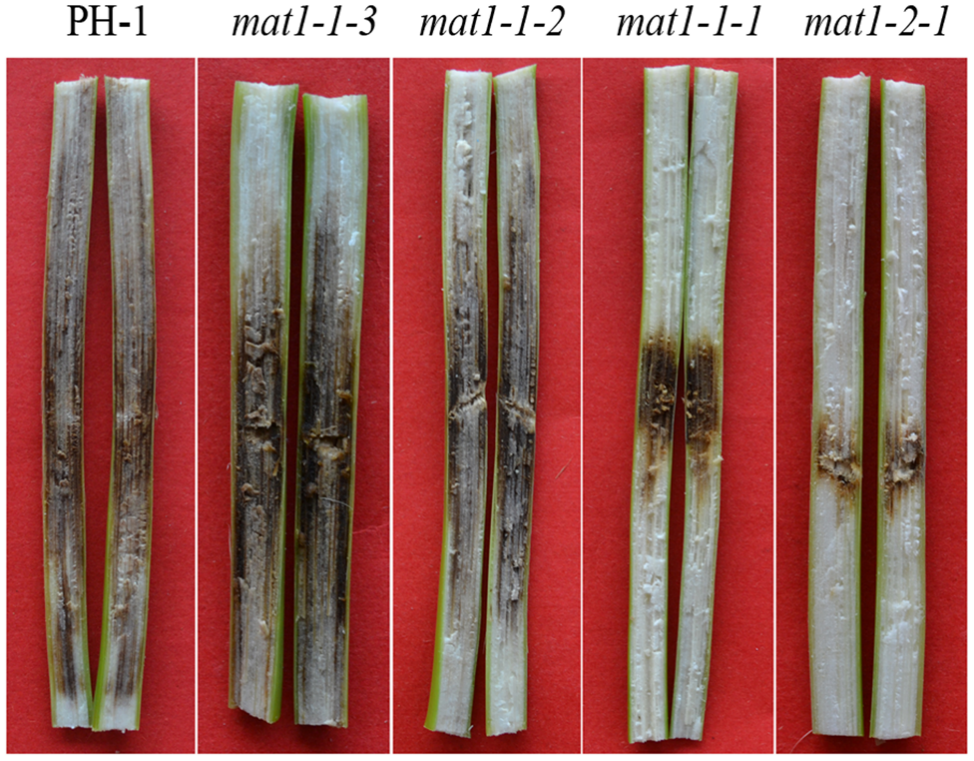
*mat1-1-1* and *mat1-2-1* mutants were defective in corn stalk infection. Corn stalks inoculated with PH-1 and four *MAT* locus gene deletion mutants. Stalk rot (discoloration) was restricted in plants inoculated with the *mat1-1-1* and *mat1-2-1* mutants.

We also assayed DON production and virulence on flowering wheat heads with the *MAT* locus TF gene deletion mutants. In comparison with the wild type, none of these mutants had obvious changes in virulence in infection assays with flowering wheat heads, which was consistent with a previous report [9]. However, in infection assays with corn stalks, we noticed that the *mat1-1-1* and *mat1-2-1* deletion mutants, but not the *mat1-1-2* and *mat1-1-3* mutants, were reduced in virulence (Fig. 4). When the stalk rot areas were measured, the virulence of the *mat1-1-1* and *mat1-2-1* deletion mutants were reduced 66% and 77% in comparison with that of the wild type. In nature, corn also is a host plant for *F.* graminearum, which often produces abundant perithecia on corn stalks. The defects of the *mat1-1-1* and *mat1-2-1* deletion mutants in corn stalk rot assays suggest that the mating type locus may play a host-specific role in plant infection.

### Mat1-1-2 Interacts with All other*MAT* Locus TF Genes in Yeast Two-hybrid Assays

**Figure 5:**
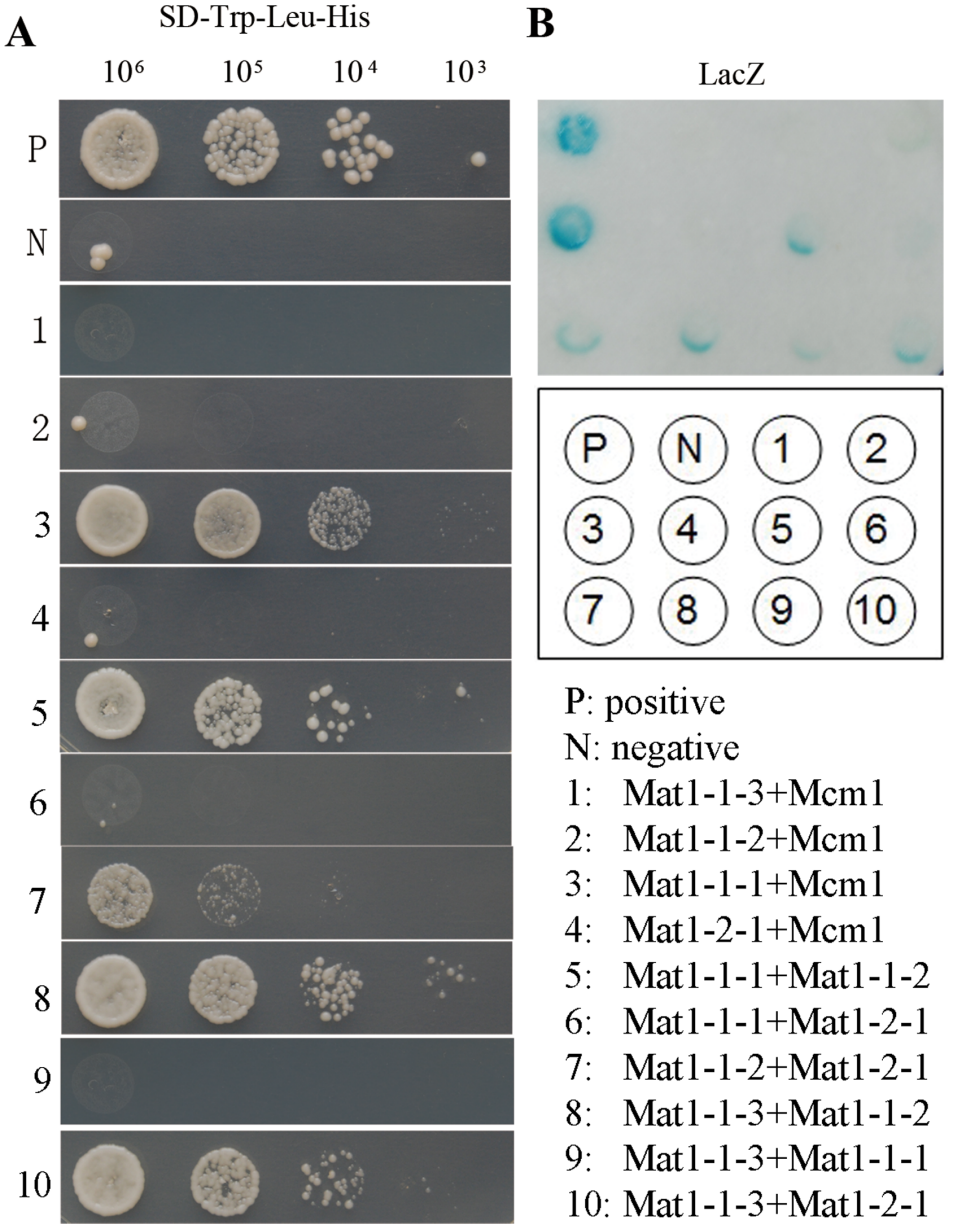
Yeast two-hybrid assays for the interactions among four*MAT* locus TFs. **A** ****. Different concentrations of yeast cells (cells/ml) of the transformants expressing the labeled bait and prey constructs were assayed for growth on SD-Leu-Trp-His plates. P and N were the positive and negative controls provided in the BD Matchmaker library construct kit. **B**. The same set of yeast transformants was assayed for β-galactosidase activities.

Because of similar defects of the *MAT* TF mutants in sexual reproduction, it is likely that these *MAT* locus genes interact with each other and function in a protein complex (similar to the Mat*a*-Matα interaction in *S. cerevisiae*) during sexual reproduction or ascosporogenesis inside perithecia. To test this hypothesis, we amplified the cDNA clones of all the *MAT* locus genes and assayed for their pair-wise interactions. In yeast two-hybrid assays, Mat1-1-1 interacted with Mat1-1-2 but not with Mat1-2 or Mat1-1-3. Mat1-1-2 and Mat1-1-3 interacted with each other and both of them interacted with Mat1-2 (Fig. 5). Although it is surprising that the direct interaction between Mat1-1-1 and Mat1-2 was not observed, Mat1-1-2 interacted with all the other *MAT* locus TF proteins, which may bring Mat1-1-1 and Mat1-2 together in a protein complex in *F. graminearum*.

In attempt to further characterize the interactions among the *MAT* locus genes, we generated the BiFC constructs and transformed them in pairs into PH-1. In the resulting transformants (Table 1), we assayed for YFP signals in conidia, germlings, hyphae, and perithecium tissues. Unfortunately, we failed to observe obvious YFP signals in repeated experiments in all the resulting transformants, including the *MAT1-1-2*-YFPC *MAT1-1-1*-YFPN transformant (Table 1). In addition to the possibility that these *MAT* locus genes are expressed at a relatively low level, their YFP fusion proteins may be unstable or their interactions are too transient.

### Mat1-1-1 Interacts with FgMcm1

**Figure 6:**
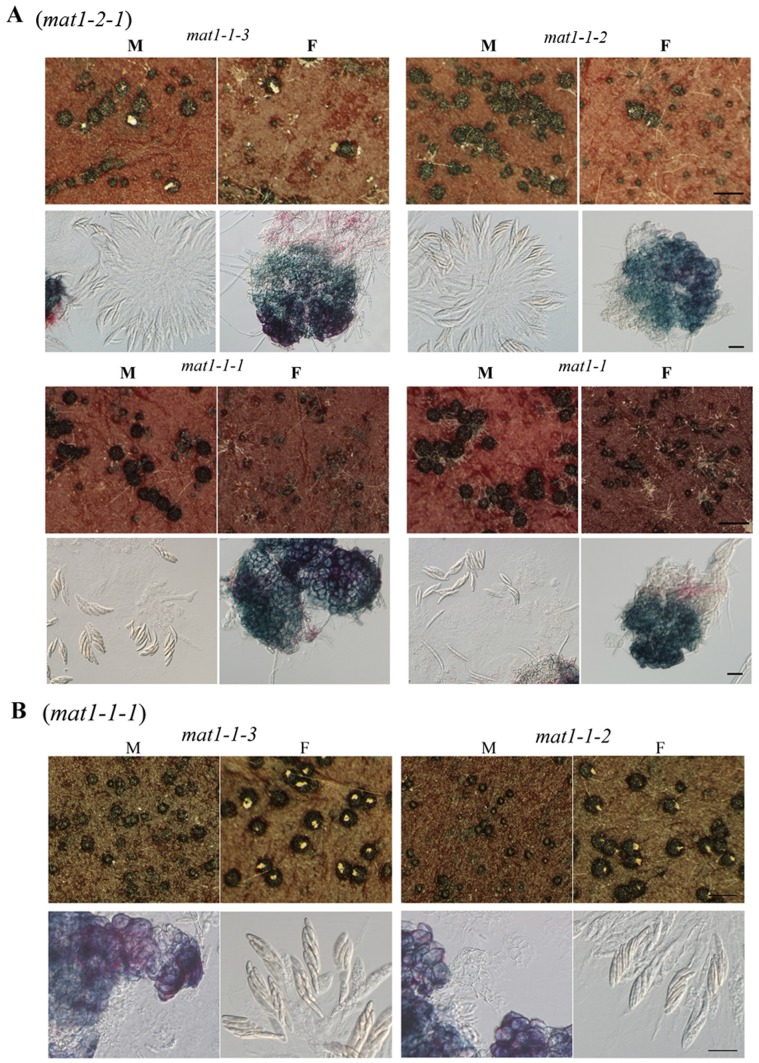
Outcrossing defects in the*mat1-2-1* and various *mat1-1* mutants. **A** ****. The *mat1-2-1* mutant was crossed as the male (M) or female (F) with the *mat1-1-3*, *mat1-1-2*, *mat1-1-1*, and *mat1-1* mutants. **B.** The *mat1-1-1* mutant was crossed as the male (M) or female (F) with the *mat1-1-3* and *mat1-1-2* mutants. The upper panels showed the size of representative perithecia and production of ascospore cirrhi (bar = 1 mm). The lower panels showed perithecia with or without fascicles of asci (bar = 20 µm).

In the budding yeast, Mcm1 interacts with Mata or Matα to regulate different processes in haploid or diploid cells [25], [26]. We also assayed the interaction of different *MAT* locus genes with the orthologs of Mcm1 in *F. graminearum.* In yeast two-hybrid assays, only Mat1-1-1 interacted with FgMcm1 (Fig. 6). Although Mat1-1-2 interacted with all other *MAT* locus genes, it did not interact with FgMcm1.

### *MAT1-1-1* and *MAT1-2-1* Play Different Roles in Outcrosses

Although it is a homothallic fungus, out-crossing is possible in *F. graminearum*. In the perithecia formed by the *mat1-1-2* (male) and *mat1-1-3* (female) or the reciprocal crosses, normal perithecia and ascospore cirrhi were produced, indicating that these two mutants were not defective in male or female fertility and *MAT1-1-2 mat1-1-3*/*mat1-1-2 MAT1-1-3* heterozygous dikaryotic or diploid cells were normal in the development of asci and ascospores.

In crosses with the *mat1-1-2* and *mat1-1-3* mutants, fertile perithecia were formed only when the *mat1-1-1* or *mat1-1* deletion mutant [6] was used as the female. When the *mat1-1-1* mutant was used as the male, only sterile, small perithecia were formed, suggesting that *MAT1-1-1* is required for male fertility but dispensable for female fertility (Table 2; Fig. 6). In contrast, *MAT1-2-1* may play a more important role in female fertility than in male fertility because the *mat1-2-1* deletion mutant displayed a female-specific defect in crosses with the *mat1-1-2* and *mat1-1-3* mutants (Table 2; Fig. 6). In the crosses with the *mat1-1-2* and *mat1-1-3* mutants, normal, fertile perithecia were formed when the *mat1-2-1* mutant was used as the male. When the *mat1-2-1* mutant was used as the female, most of the perithecia, similar to those of the *mat1-2-1* mutant formed in self-crosses, were small and sterile. However, approximately 5% of them were similar to the wild-type perithecia in size and produced normal ascospores. These results indicate that *MAT1-2-1* is not essential but important for female fertility in *F. graminearum*.

**Table 2:** Defects of different mutants in outcrossing.

Female strain*	Male*	Perithecium and ascospore formation
*mat1-1-1* mutant	*mat1-1-2*	Normal
	*mat1-1-3*	Normal
	*mat1-2-1*	Partially normal perithecia, defective in ascospore releasing
*mat1-1-2* mutant	*mat1-1-1*	Small, sterile perithecia
	*mat1-1-3*	Normal
	*mat1-2-1*	Normal
*mat1-1-3* mutant	*mat1-1-1*	Small, sterile perithecia
	*mat1-1-2*	Normal
	*mat1-2-1*	Normal
*mat1-2-1* mutant	*mat1-1-1*	Small, sterile perithecia
	*mat1-1-2*	Rare
	*mat1-1-3*	Rare
	*mat1-1*	Partially normal perithecia, defective in ascospore releasing
*mat1-1* mutant	*mat1-2-1*	Small, sterile perithecia

* Carrot agar (CA) cultures fertilized with conidia harvested from CMC cultures. Perithecium development and ascospore formation were examined 10 days post-perithecial induction.

When the *mat1-2-1* mutant was crossed as the female with the *mat1-1-1* mutant, only smaller, sterile perithecia were formed (Table 2; Fig. 6). We failed to observe perithecia with ascospores in repeated experiments. These results were consistent with the defects of the *mat1-2-1* and *mat1-1-1* mutants in female and male fertility, respectively (Fig. 6). However, perithecia with ascospore cirrhi were produced by the *mat1-1-1* (female) x *mat1-2-1* (male) cross (Fig. 6). Therefore, the *mat1-1-1 MAT1-2-1*/*MAT1-1-1 mat1-2-1* dikaryotic or diploid cells were normal in later stages of perithecium development and ascosporogenesis. These results further indicate that the *MAT1-2-1* and *MAT1-1-1* genes play critical roles in earlier stages of perithecium development.

### The*FgSO* Gene and Hyphal Fusion are Required for Sexual Reproduction

**Figure 7:**
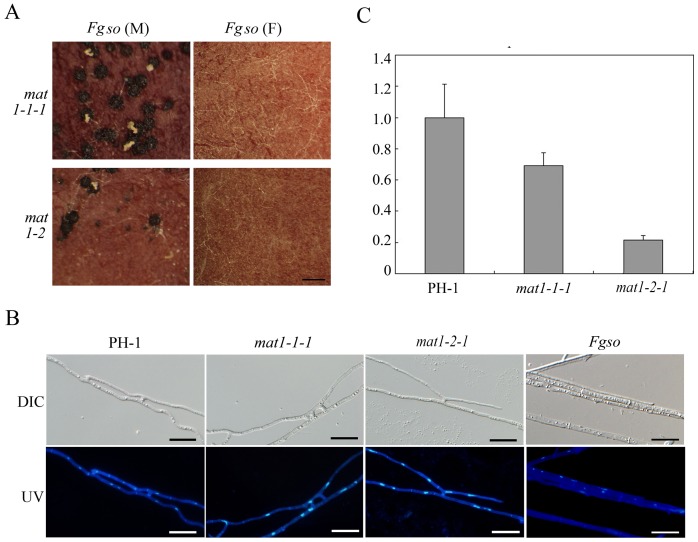
Mating defects of the*Fgso* mutant. **A** ****. The *Fgso* mutant was crossed as the male (M) or female (F) with the *mat1-1-1* and *mat1-2-1* mutants. Although it was female sterile, the *Fgso* mutant retained male fertility. Bar = 1 mm. **B**. Assays for hyphal fusion in carrot agar cultures of the wild type (PH-1) and the *mat1-1-1, mat1-2-1,* and *Fgso* mutants. **C**. The expression level of *FgSO* in the wild type and the *mat1-1-1* and *mat1-2-1* mutants.

Because the *SO* gene is known to regulate hyphal fusion and female fertility in *N. crassa* [27], [28], we generated the *Fgso* deletion mutant in *F. graminearum* by the split marker approach (Fig. S5). The *Fgso* mutant was sterile in self-crosses. When crosses with the *mat1-1-1* and *mat1-2-1* mutants, the *Fgso* mutant was female sterile but retained male fertility (Fig. 7A). Fertile perithecia and normal ascospores were observed only in crosses with the *mat1-1-1* and *mat1-2-1* mutants when the *Fgso* mutant functioned as the male. The *Fgso1* (male) x *mat1-2-1* (female) cross produced fewer fertile perithecia than the *Fgso1* (male) x *mat1-1-1* (female) cross, which is consistent with the female-specific defects of the *mat1-2-1* mutant.

Similar to the *so* mutant in *N. crassa*, the *Fgso* deletion mutant was defective in hyphal fusion (Fig. 7B). In comparison with the wild type, hyphal fusion was normal in the *mat1-1-1* mutant (Fig. 7B). In the *mat1-2-1* deletion mutant, hyphal fusion still occurred but at a reduced frequency (Fig. 7B). When assayed by qRT-PCR, the expression level of *FgSO* was significantly reduced in the *mat1-2-1* mutant but not in the *mat1-1-1* mutant (Fig. 7C). Therefore, the defects of the *mat1-2-1* mutant in hyphal fusion may be directly related to the down-regulation of *FgSO* expression. *MAT1-2-1* may be involved in regulating the expression of *FgSO* during sexual reproduction in *F. graminearum*.

### Deletion of one*MAT* Gene Affects the Expression of Other *MAT* Locus Genes

**Figure 8:**
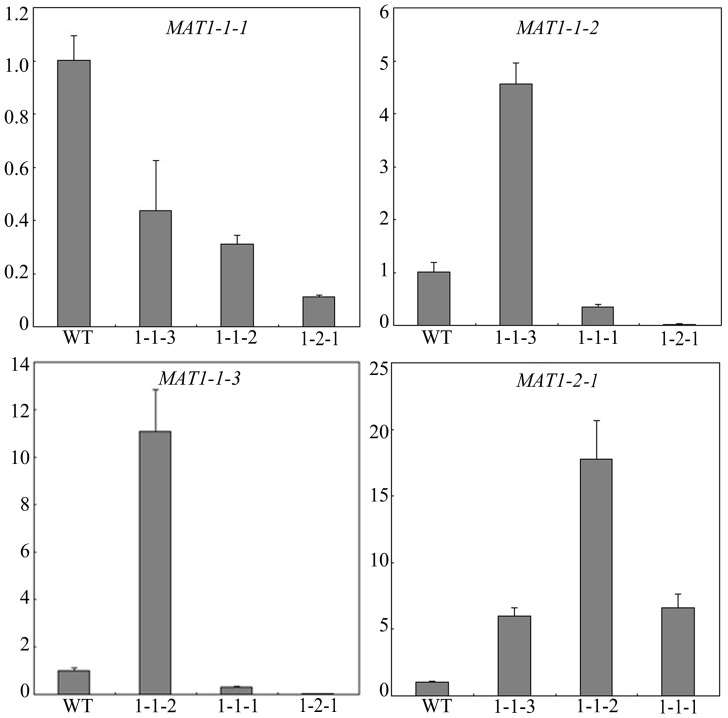
The expression of other*MAT* locus genes in the *mat1-2-1* and various *mat1-1* mutants. The expression of *MAT1-1-1*, *MAT1-1-2*, *MAT1-1-3*, and *MAT1-2-1* in PH-1 (WT) or the *mat1-1-1, mat1-1-2, mat1-1-3,* and *mat1-2-1* deletion mutants. The relative expression level of each gene in the wild type was arbitrarily set to 1. RNA samples were isolated from aerial hyphae harvested from 7-day-old carrot agar cultures. Mean and standard error were calculated from three independent biological replicates.

One possible explanation for similar phenotypes of the *MAT* locus gene deletion mutants is that deletion of any one of them may affect the expression of the others. To test his hypothesis, we isolated RNA from different mutants and assayed for the expression levels of individual *MAT* locus TF genes by qRT-PCR (Fig. 8). *MAT1-1-1* expression was significantly reduced in all the *MAT* locus gene deletion mutants assayed in comparison with the wild type, which may be related to similar mating defects in self-crosses of these mutants because *MAT1-1-1* is important for male fertility. The expression of *MAT1-1-2* and *MAT1-1-3* was up-regulated in the *mat1-1-3* and *mat1-1-2* mutants, respectively (Fig. 8). Thus, we concluded that deletion of one of these two genes increased the transcription of the other, which may be related to the fact that they share the same bidirectional promoter. Both *MAT1-1-2* and *MAT1-1-3* were down-regulated in the *mat1-1-1* and *mat1-2-1* mutants (Fig. 8), indicating that their expression may be positively regulated by *MAT1-1-1* and *MAT1-2-1*, two *MAT* locus genes with up-regulated expression during earlier stages of sexual reproduction (Fig. 1B). Because their close proximity, it is possible that deletion of individual *MAT* locus genes affect the expression of nearby genes by altering chromatin structures or promoter activities. The other possibility is that lack of one mating type protein may affect the expression of other *MAT* genes because they may interact with each other to form a protein complex in *F. graminearum.*

### Increased Expression of Pheromone or Pheromone Receptor Genes in the*MAT* Locus Gene Mutants

**Figure 9:**
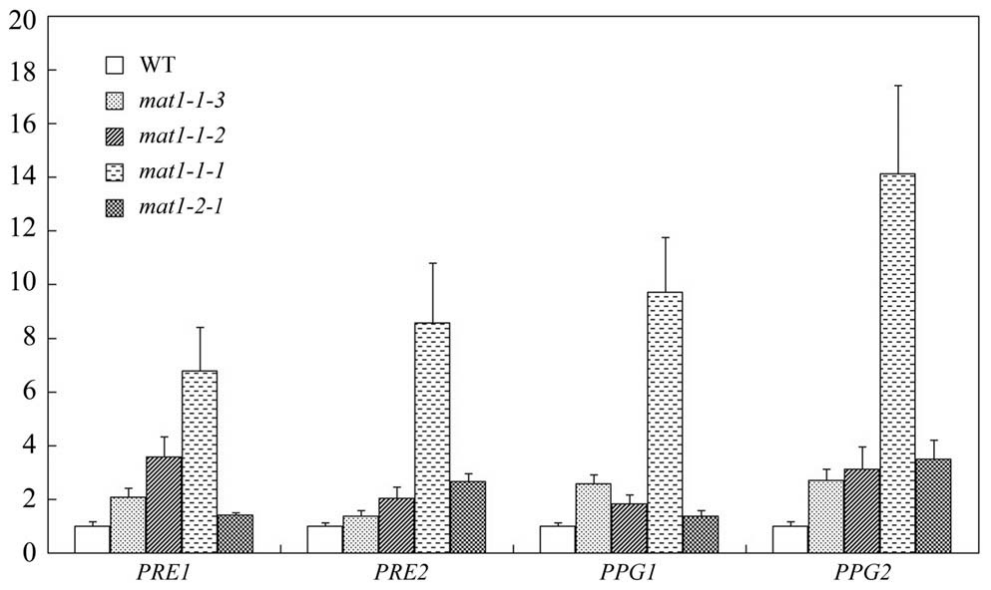
Effects of*MAT* locus gene deletion on the expression of pheromone and pheromone receptor genes. The expression levels of *PRE1*, *PRE2, PPG1*, and *PPG2* in PH-1 or the *mat1-1-*3, *mat1-1-2, mat1-1-1,* and *mat1-2-1* deletion mutants were assayed by qRT-PCR. The relative expression level of each gene in the wild type was arbitrarily set to 1. RNA samples were isolated from aerial hyphae harvested from 7-day-old carrot agar cultures. Mean and standard error were calculated from three independent biological replicates.

*PRE1, PRE2, PPG1,* and *PPG2* are the pheromone and pheromone receptor genes in *F. graminearum* [29], [30]. When assayed by qRT-PCR with RNA isolated from hyphae grown on 7-day-old carrot agar cultures, all the *PRE* and *PPG* genes were up-regulated in individual *MAT* locus gene deletion mutants (Fig. 9). In the *mat1-1-1* mutant, the expression level of *PRE1, PRE2, PPG1,* and *PPG2* was increased 7-, 9-, 10-, and 14-fold, respectively. The up-regulation of these pheromone and pheromone receptor genes varied between 1.7- and 3.8-fold in the other *MAT* locus gene deletion mutants (Fig. 9). These results indicate that *MAT1-1-1*, and possibly other *MAT* locus genes, negatively regulate the expression of the *PRE1, PRE2, PPG1,* and *PPG2* genes during vegetative growth in *F. graminearum*.

### Constitutive Expression of*MAT1-2-1* Affects Sexual Reproduction

**Figure 10:**
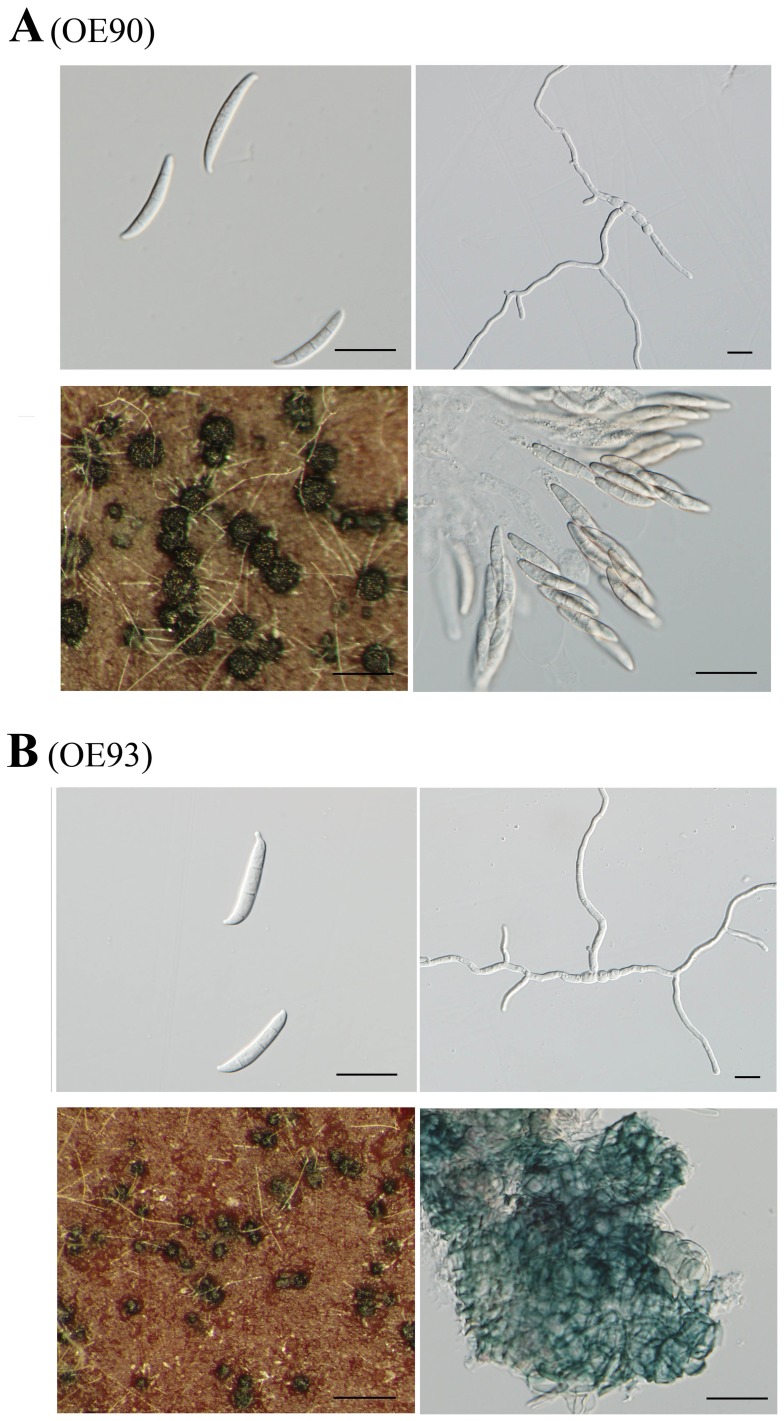
Vegetative growth and sexual reproduction in the P_TrpC_-*MAT1-1-3* and P_TrpC_-*MAT1-2-1* transformants. **A** ****. Conidia and germ tubes of the P_TrpC_-*MAT1-1-3* (OE90) and P_TrpC_-*MAT1-2-1* (OE93) transformants. Bar = 20 µm. **B**. Carrot agar cultures (bar = 1 mm) and cracked perithecia (bar = 20 µm) of transformants OE90 and OE93. Sterile perithecia formed by the P_TrpC_-*MAT1-2-1* transformant were smaller than those of the P_TrpC_-*MAT1-1-3* transformant.

The expression of *MAT* locus genes are repressed in vegetative hyphae in *F. graminearum*. To determine the effects of improper expression of *MAT1-2-1* and *MAT1-1-3*, we used the constitutive TrpC promoter [31] to express these two *MAT* locus TF genes. The P_TrpC_-*MAT1-2-1* and P_TrpC_-*MAT1-1-3* constructs were transformed into PH-1. The resulting transformants were normal in vegetative growth and asexual reproduction although GFP signals were observed in vegetative hyphae (Fig. 2B) and conidia (Fig. S6). Whereas the P_TrpC_ *-MAT1-1-3* transformant had no obvious defects in sexual reproduction, the P_TrpC_ *-MAT1-2-1* transformant produced perithecia without ascospores (Fig. 10), indicating that constitutive expression of *MAT1-2-1* negatively impacted ascospore formation in *F. graminearum*.

## Discussion

In *F. graminearum*, the *MAT1-1-3* and *MAT1-1-2* genes are likely under the control of the 259-bp bidirectional promoter and they had a similar expression profile during sexual reproduction (Fig. 1B). In *N. crassa*, the *mata*-1 gene encodes a protein with the core binding site of CTTTG [24]. The palindromic GAAAGCTTTC sequence in this bidirectional promoter region has two CTTTG-like sequences in different strands. *MAT1-2-1*, a homolog of *mata-1*, may have similar recognition site in *F. graminearum* and bind to this palindromic sequence to regulate the expression of *MAT1-1-3* and *MAT1-1-2*. Other transcription factors, such as *Mcm1*, may also bind to this bidirectional promoter region to negatively regulate the expression of *MAT1-1-3* and *MAT1-1-2* during vegetative growth or positively regulate their expression during sexual reproduction. The 259-bp promoter region has two sequences similar to the consensus Mcm1-binding site CCNNNWWRGG (rulai.cshl.edu/cgi-bin/SCPD/getfactor?MCM1).

During the preparation of this manuscript, a paper on functional characterization of the expression and knockout mutants of individual *MAT* locus genes in *F. graminearum* was published [9]. In this study, fungal biomass used for RNA isolation were harvested by directly scraping off hyphae and fruiting bodies together from the surface of mating cultures at 1, 4, 7, and 10 days post-perithecial induction (dpi). We found that the expression of *MAT1-1-1* and *MAT1-2-1* peaked at 1 dpi but *MAT1-1-2* and *MAT1-1-3* had the highest expression level at 4 dpi. Kim and colleague conducted qRT-PCR analysis with RNA isolated from mating cultures at 2, 4, 6, 8, 10, and 12 dpi [9]. Although there were slight differences in the expression levels of individual *MAT* genes among different strains of *F. graminearum*, they found that the expression of *MAT1-1-1* and *MAT1-2-1* peaked at 2 dpi and *MAT1-1-2* and *MAT1-1-3* had the highest expression level at 4 dpi in PH1 [9], which is similar to the overall expression profiles of these four *MAT* TFs reported in this study. However, the exact fold changes for individual *MAT* TF genes were different between what was reported recently [9] and our observations, which may be related to different methods used to harvest fungal biomass for RNA isolation. We noticed that Kim and colleagues separated perithecia from hyphae [9]. Because perithecium development is not synchronous, our approach may have perithecia of different development stages mixed with undifferentiated hyphae. In addition, we used 0.1% Tween 20 instead of 2.5% Tween 60 to induce sexual reproduction by pressing down aerial hyphae (Kim et al., 2012).

In this study, the expression levels of *PPG1*, *PPG2*, *PRE1,* and *PRE2* in mutants deleted of individual *MAT* TF genes were assayed with RNA samples isolated from 7-day-old carrot agar cultures before perithecial induction. We found that the expression of all the *PRE* and *PPG* genes was significantly up-regulated in the *mat1-1-1* mutant (Fig. 9). Their expression levels also were increased in the other *MAT* TF deletion mutants but not as significantly as in the *mat1-1-1* mutant (Fig. 9). These results suggested that deletion of individual *MAT* TF genes may stimulate the expression of *PPG* and *PRE* genes in vegetative hyphae of *F. graminearum*. In *F. graminearum*, it has been reported that the *PPG* and *PRE* genes are not essential for sexual reproduction [30]. Whereas the expression of *PPG2* increased at different stages of perithecial development in the *mat1-1*deletion mutant, up-regulation of *PPG1, PRE1,* and *PRE2* was not observed in the *mat1-1* and *mat1-2* deletion mutants. Whereas Kim and colleagues [30] assayed the expression levels of the *PPG* and *PRE* genes in cultures after sexual induction, we assayed their expression in vegetative hyphae. Also, we used mutants deleted of individual *MAT* TF genes instead of the *mat1-1* and *mat1-2* locus deletion mutants.

One of our original goals was to determine the expression and subcellular localization of these four *MAT* locus genes during the switching from vegetative growth to sexual reproduction. Therefore, we generated knock-in GFP-fusion transformants in this study. Unfortunately, GFP signals were too faint or not detectable in the knock-in GFP fusion transformants of these *MAT* locus genes. The *MAT1-1-3* knock-in transformant had the strongest GFP signals but we failed to detect fluorescence signals in the nucleus during sexual reproduction. One likely explanation is that their expression at the protein level was relatively low and perithecium tissues had a strong fluorescence background. It is also possible that *MAT* locus proteins only localize to the nucleus transiently. Interestingly, GFP signals were observed in the nucleus in vegetative hyphae and conidia but not in perithecium tissues of the P_TrpC_-*MAT1-2-1*-GFP transformant. In the P_TrpC_-*MAT1-1-3*-GFP, GFP signals in vegetative hyphae were stronger than the GFP knock-in transformant but were not localized to the nucleus (Fig. 2B).

Similar to what were reported [9], the *mat1-1-1, mat1-1-2,* and *mat1-1-3* mutants produced smaller perithecia with thicker perithecium walls than the wild type but did not form asci or ascospores in self-crosses, indicating that all these four genes in the *MAT* loci are required for ascospore production. Unlike the *mat1-2-1* mutant of GZ3639 [9], although it was reduced in perithecium formation, the *mat1-2-1* mutant generated in this study still produced numerous small, sterile perithecia. Close examination revealed that ascogenous hyphal growth inside perithecia was blocked or defective in the *mat1-2-1* and other *MAT* locus gene deletion mutants (Fig. 3).

In out-crosses, we found that the *mat1-1-1* mutant displayed male-specific defects in mating although it was normal in female fertility. Kim and colleagues did not assay the defects of these *MAT* locus gene mutants in outcrosses [9]. To our knowledge, no other genes are known to be essential for male fertility in *F. graminearum*. In *M. oryzae*, the *mcm1* mutant was reported to be male sterile and it was blocked in the production of microconidia [32]. *F. graminearum*, unlike many other Fusarium species, does not produce microconidia. It will be important to determine the role of *MAT1-1-1* in male fertility.

Interestingly, the *mat1-2-1* deletion mutant displayed a female-specific defect in the production of normal perithecia and ascospores in out-crosses. Most of the perithecia produced in crosses with the *mat1-2-1* mutant as the female were small and sterile. Although a few of them were fertile, they failed to produce cirrhi. Therefore, *MAT1-2-1* is not essential but plays an important role in perithecium development, likely before the formation of dikaryotic hyphae or diploid cells. There are many mutants known to be defective in female fertility in *F. graminearum*. Most of them are blocked in the production of proto-perithecia, including the *mgv1* and *Gpmk1* mutants [33], [34], [35], [36]. The *MAT1-1-2* and *MAT1-1-3* genes were dispensable for mating either as the male or female in out-crosses. It is possible that *MAT1-1-2* and *MAT1-1-3* are functionally redundant. It will be of interest to generate the *mat1-1-2 mat1-1-3* double mutant and determine its defects in outcrosses with other strains.

In yeast, *MATa* interacts with *MATα* in diploid cells to suppress the expression of haploid-specific genes [14], [25]. In *F. graminearum*, the dikaryotic and diploid stages are transient and occur only inside perithecia. In yeast two-hybrid assays, we observed the interaction of *MAT1-1-2* with all other *MAT* locus genes. The interactions of *MAT1-1-3* with *MAT1-1-1* and *MAT1-2-1* also were detected. To detect the interaction of different *MAT* locus genes by BiFC assays, we generated the transformants expressing different YFPC- and YFPN-fusion constructs (Table 1). Unfortunately, we failed to observe YFP signals in any of these transformants during vegetative growth or sexual reproduction. The expression levels of these *MAT* locus genes may be too low and fluorescence background may be too high in perithecium tissues. It is also possible that the interactions among the *MAT* locus TF genes may be too transient to be detected by BiFC assays.

In *N. crassa*, *SO* is important for hyphal fusion. The *so* mutant displayed pleiotropic defects in growth, conidiation, and sexual reproduction [27], [28]. Orthologs of *SO* are well conserved in filamentous ascomycetes but it is absent in yeast and its function in plant pathogenic fungi is not clear. In *F. graminearum*, the *Fgso* mutant was defective in hyphal fusion and sterile in self-crosses. It was female sterile but male fertile in outcrosses. In infection assays, the *Fgso1* mutant was significantly reduced in virulence. Because *FgSO* expression and hyphal fusion were reduced in the *mat1-2-1* mutant, it is possible that *MAT1-2-1* may directly or indirectly regulate the expression of *FgSO* in *F. graminearum*.

Interestingly, although the *MAT* locus gene deletion mutants were normal in wheat head infection, we noticed that the *mat1-1-1* and *mat1-2-1* mutants, but not the *mat1-1-2* and *mat1-1-3* mutants, were reduced in virulence in corn stalk rot assays. The same results were obtained in repeated experiments. The *mat1-1-1* and *mat1-2-1* mutants may be defective in adaptation to or colonization of corn stalks, which are substrates for *F. graminearum* to produce perithecia in the field. It will be important to further characterize the defects of the *mat1-1-1* and *mat1-2-1* mutants in corn stalk infection and determine the underlying mechanism. Corn stalks are known to accumulate diterpenoid phytoalexins [37]. The *mat1-1-1* and *mat1-2-1* mutants may have enhanced sensitivity to these phytoalexins or may be defective in overcoming other plant defensive responses in corn stalks.

## Materials and Methods

### Strains and Culture Conditions

The wild-type strain and mutants of *F. graminearum* used in this study are listed in Table 1. Cultures were routinely grown on PDA plates at 25°C [38], [39]. Conidiation in 5-day-old CMC cultures and growth rate on PDA plates were measured as described [40], [41]. For DNA extraction, vegetative hyphae were harvested from 2-day-old YEPD (1% yeast extract, 2% peptone, 2% glucose) cultures. For genetic crosses, aerial hyphae of 7-day-old carrot agar cultures were pressed down with 300 µl of sterile 0.1% Tween 20 or conidium suspensions (10^5^ conidia/ml) of the male strains and incubated under black light [42]. Perithecium formation and cirrhi production were assayed after incubation at 25°C for 2 weeks. Protoplast preparation and fungal transformation were performed as described [33], [43], [44]. For transformation, hygromycin B (Calbiochem, La Jolla, CA) and geneticin (Sigma-Aldrich, St. Louis, MO) were added to the final concentration of 250 and 150 µg/ml, respectively [45].

### Plant Infection Assays

For wheat head and corn stalk infection assays, freshly harvested conidia were re-suspended to 10^5^ spores/ml in sterile distilled water. Flowering wheat heads of cultivar Xiaoyan22 were inoculated with 10 µl of conidium suspensions at the fifth spikelet from the base of the spike and scored for head blight symptoms as described [46], [47]. Stalks of 8-week-old corn plants of cv. Pioneer 2375 were inoculated with toothpicks dipped in conidium suspensions as described [41], [48]. Stalk rot symptoms were examined after splitting the stalks longitudinally along the inoculation site 14 dpi.

### qRT-PCR Analysis

RNA samples were isolated from conidia, vegetative hyphae, and fungal biomass (hyphae and fruiting bodies) harvested from carrot agar cultures by scraping gently with a spatula with the TRIzol reagent (Invitrogen, Carlsbad, CA). For each experiment, at least three independent biological replicates were conducted. First-strand cDNA was synthesized with the Fermentas 1st cDNA synthesis kit (Hanover, MD) following the instructions provided by the manufacturer. For the internal control, the *FgTUB2* beta-tubulin gene of *F. graminearum* was amplified with primers TubQF and TubQR [47].

### Generation of the*mat1-1-1*, *mat1-1-2*, *mat1-1-3*, *mat1-2-1*, and *Fgso* Mutants

The split-marker approach was used to generate the gene replacement constructs for the *MAT* locus and *FgSO* genes (Fig. S3; Fig. S5). Putative knockout mutants were identified by PCR and confirmed by Southern blot hybridizations to confirm the gene replacement event. All of the mutants generated in this study were preserved in 15% glycerol at −80°C.

### Generation of GFP knock-in Transformants and GFP Fusion Constructs

A modified split-marker approach was used to generate the GFP knock-in transformants (Fig. S1). Primers 1F/2R and 3F/4R were used to amplify the flanking sequences of the stop codons of the target genes (Fig. S1). The GFP and geneticin resistance marker (Gen^R^) fusion construct pGTP was generated in this study by overlapping PCR and used as the split marker for transformation. The resulting G418-resistant transformants were screened by PCR. In-frame fusions of GFP with *MAT* locus genes were then confirmed by sequencing analysis with PCR products.

To generate the *MAT1-1-3-*GFP and *MAT1-2-1-*GFP fusion constructs, PCR products containing the genomic fragments of the target genes were amplified and cloned into pFL2 [49] by the yeast gap repair approach [50]. All GFP fusion constructs were verified by sequencing analysis and transformed into protoplasts of the corresponding mutants. G418-resistant transformants harboring the transforming constructs were identified by PCR and confirmed by the presence of GFP signals.

### Yeast Two-hybrid Assays

Protein-protein interactions were assayed with the Matchmaker yeast two-hybrid system (Clontech, Mountain View, CA). ORFs of the *MAT1-1-2, MAT1-1-3, MAT1-2-1*, and *MCM1* were amplified from first-strand cDNA of PH-1 and cloned into pGBK7 (Clontech) as the bait constructs. For the *MAT1-1-1, MAT1-2-1,* and *MCM1* genes, their ORFs were amplified and cloned into pGADT7 as the prey constructs. The resulting bait and prey vectors were co-transformed in pairs into yeast strain AH109 (Clontech). The Leu+ and Trp+ transformants were isolated and assayed for growth on SD-Trp-Leu-His medium and galactosidase activities with filter lift assays as described [51]. The positive and negative controls were provided in the Matchmaker library construction kit (Clontech).

### BiFC Assays

Plasmids pHZ65 and pHZ68 containing the N-terminal (1-154aa) and C-terminal (155-238 aa) regions of YFP [52], respectively, were used to generate the YFPN- or YFPC-fusion constructs. The *MAT1-1-1*-YFPN fusion construct was generated by cloning the *MAT1-1-1* gene amplified with primers M1native and M1YFP into pHZ65 by yeast gap repair [50]. A similar approach was used to generate the YFPN- or YFPC-fusion constructs of *MAT1-1-2*, *MAT1-1-3,* and *MAT1-2-1*. Pairs of the resulting BiFC vectors were then transformed into protoplasts of PH-1. The resulting transformants (Table 1) were screened by PCR for the presence of the target YFPN- or YFPC-fusion constructs.

## Supporting Information

Figure S1 **Generation of in-frame** ***MAT1-2-1*** **-GFP knock-in fusion transformants. A.** Diagram for the *MAT1-2-1* knock-in construct. The GFP-G418 resistant marker fragment from pGTP was used to replace the terminator sequence of *MAT1-2-1*. The lower panel showed PCR verification of GFP knock-in transformants (1–14). M, marker; WT, wild type. **B**. GFP signals in the *MAT1-2-1*-GFP knock-in transformant in conidia and hyphae. Bar = 20 µm.
(TIF)

Figure S2 Generation of the gene replacement mutants of four *MAT* locus TF genes. **A**. The*MAT1-1-1* locus and gene replacement construct. The*MAT1-1-1* and *hph* genes are marked with empty and black arrows, respectively. 1F, 2R, 3F, and 4R are the primers used to amplify the flanking sequences. Lower panels are Southern blots of the wild type (PH-1) and putative *mat1-1-1* mutants (M1, M2, and M3) hybridized with probe A (left) amplified with primers M15F/M16R and probe B (right) amplified with primers H852/H850. Panels **B, C,** and **D** were similar figures showing the gene replacement constructs and mutants of the *MAT1-1-2, MAT1-1-3*, and *MAT1-2-1* genes, respectively. E, *Eco*RI; K, *Kpn*I; X, *Xba*I
(TIF)

Figure S3 Three-day-old PDA cultures of the wild type and the *mat1-1-3, mat1-1-2, mat1-1-1,* and *mat1-2-1* mutants. No differences in growth or colony morphology were observed between PH-1 and the mutants.
(TIF)

Figure S4 Conidia and 12 h germ tubes of the wild type (PH-1) and the *mat1-1-3, mat1-1-2, mat1-1-1,* and *mat1-2-1* mutants. Bar = 20 µm.
(TIF)

Figure S5 **Generation of the** ***Fgso*** **deletion mutant**. **A.** The *FgSO* gene replacement construct (upper panel) and verification of the *Fgso* deletion mutants by Southern blot analysis (lower panel). Genomic DNA samples were digested with *Eco*RV (E). WT, the wild type strain PH-1. M1-M4, putative *Fgso* mutants. **B.** Three-day-old PDA cultures of PH-1 and the *Fgso* deletion mutant.
(TIF)

Figure S6 **GFP signals in the conidia of P_TrpC_-** ***MAT1-2-1*** **-GFP transformant**. The localization of GFP signals in the nucleus and cytoplasm of conidia harvested from 5-day-old CMC cultures of the P_TrpC_-*MAT1-2-1*-GFP transformant.
(TIF)
