# Correction: The *MAT* Locus Genes Play Different Roles in Sexual Reproduction and Pathogenesis in *Fusarium graminearum*

**DOI:** 10.1371/journal.pone.0131623

**Published:** 2015-07-01

**Authors:** Qian Zheng, Rui Hou, Juanyu, Zhang, Jiwen Ma, Zhongshou Wu, Guanghui Wang, Chenfang Wang, Jin-Rong Xu

There is an error in Fig 3. Images included in Fig 6 were inadvertently duplicated in Fig 3. Please view Fig 3 here.

**Fig 3:**
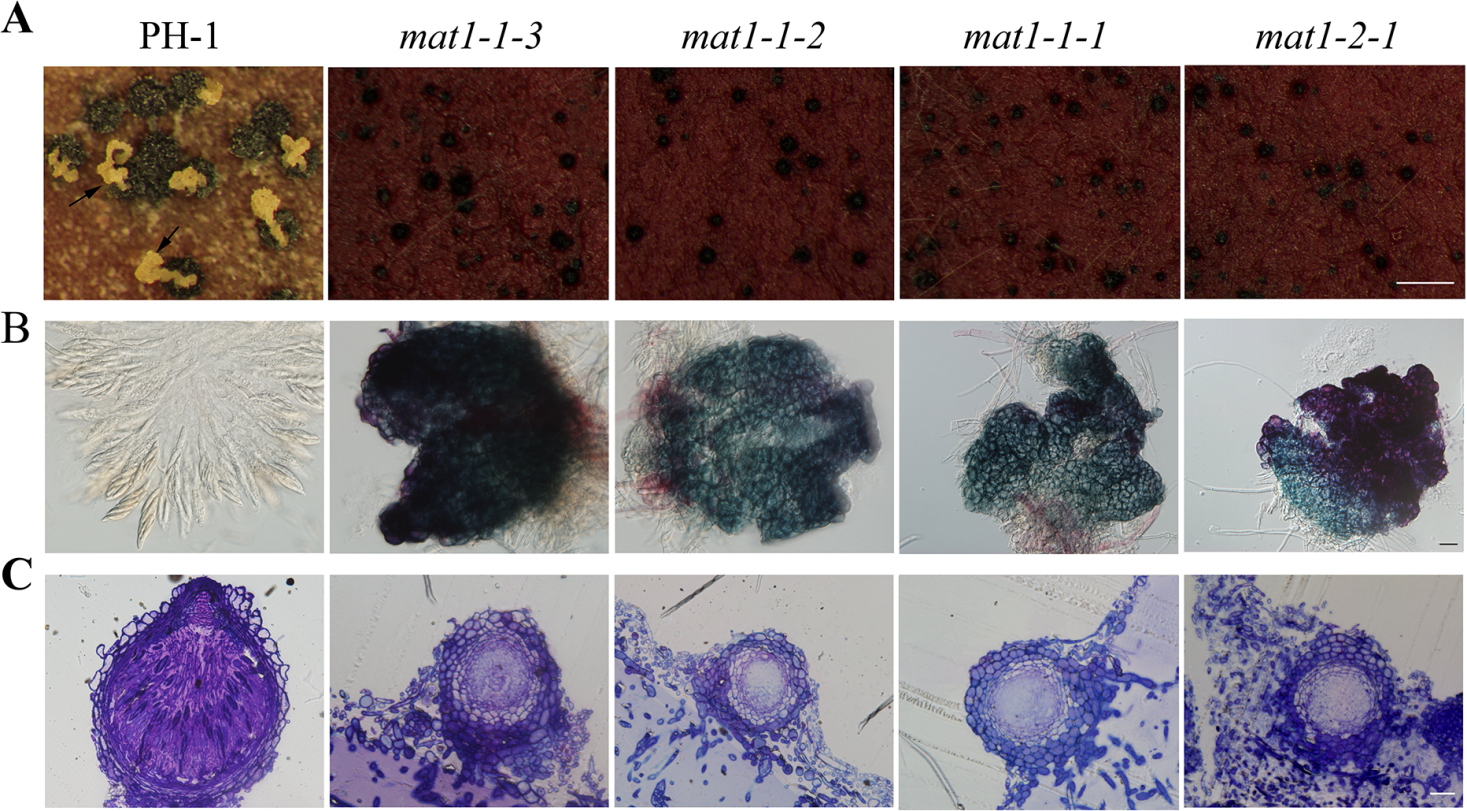
Defects of different*MAT* locus gene deletion mutants in self-crosses. **A**. Perithecia produced by 14-day-old carrot agar cultures of the wild type (PH-1) and different *MAT* locus gene deletion mutants. Arrows pointed to the cirrhi. Bar = 1 mm. **B**. Perithecia of PH-1 and the *MAT* locus gene deletion mutants were examined for ascus and ascospore development. Bar = 20 μm. **C.** Thick sections of representative perithecia produced by the wild type and mutant strains. Bar = 20 μm.
