# Supplementary figures and images for: The MAT Locus Genes Play Different Roles in Sexual Reproduction and Pathogenesis in Fusarium graminearum
Source: PLoS One. 2013 Jun 24;8(6):e66980. doi: 10.1371/journal.pone.0066980 (PMC3691137; doi:10.1371/journal.pone.0066980)

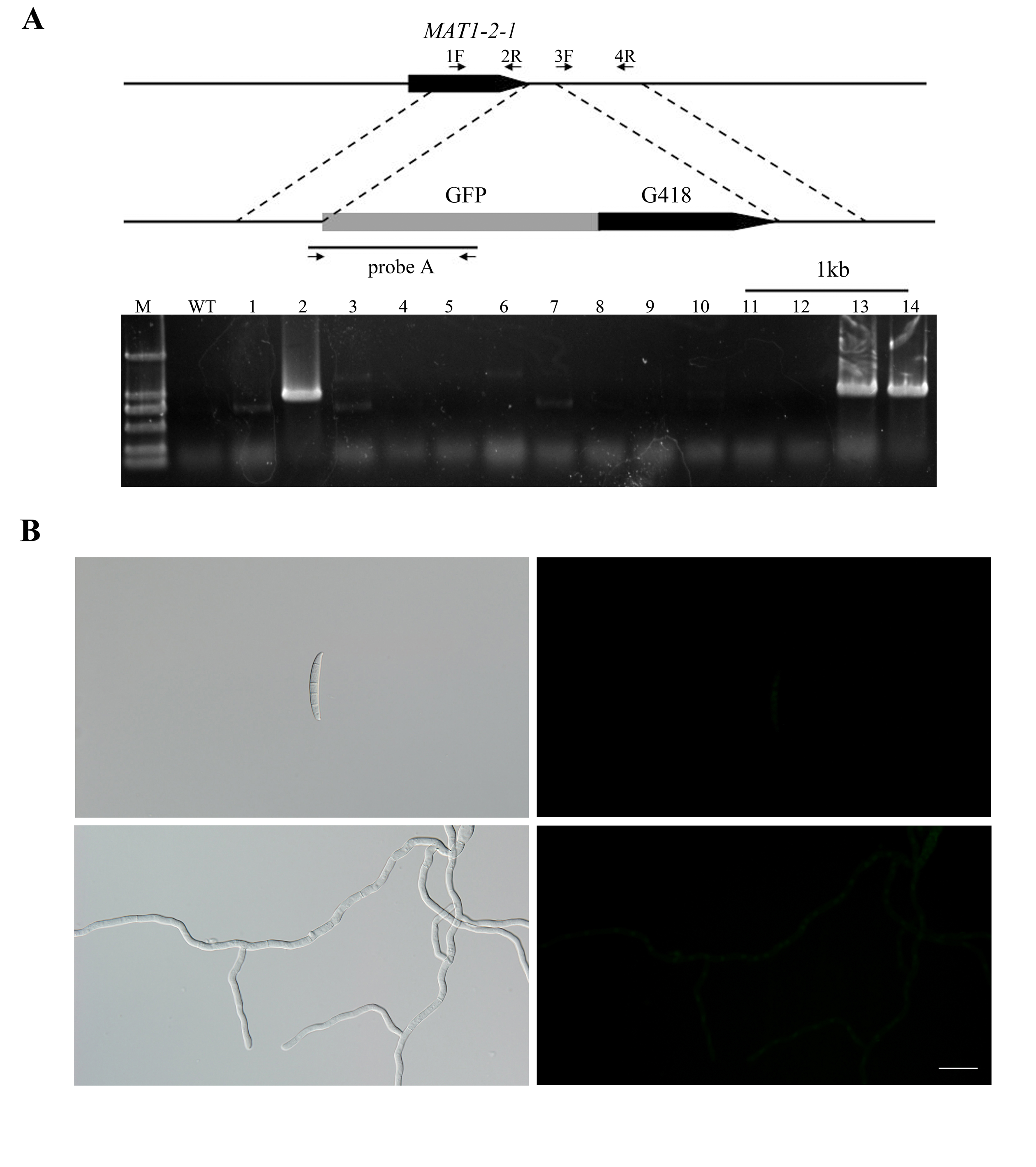

Supplement: Figure S1 — Generation of in-frame MAT1-2-1 -GFP knock-in fusion transformants. A. Diagram for the MAT1-2-1 knock-in construct. The GFP-G418 resistant marker fragment from pGTP was used to replace the terminator sequence of MAT1-2-1. The lower panel showed PCR verification of GFP knock-in transformants (1–14). M, marker; WT, wild type. B. GFP signals in the MAT1-2-1-GFP knock-in transformant in conidia and hyphae. Bar = 20 µm. (TIF) [file pone.0066980.s001.tif]

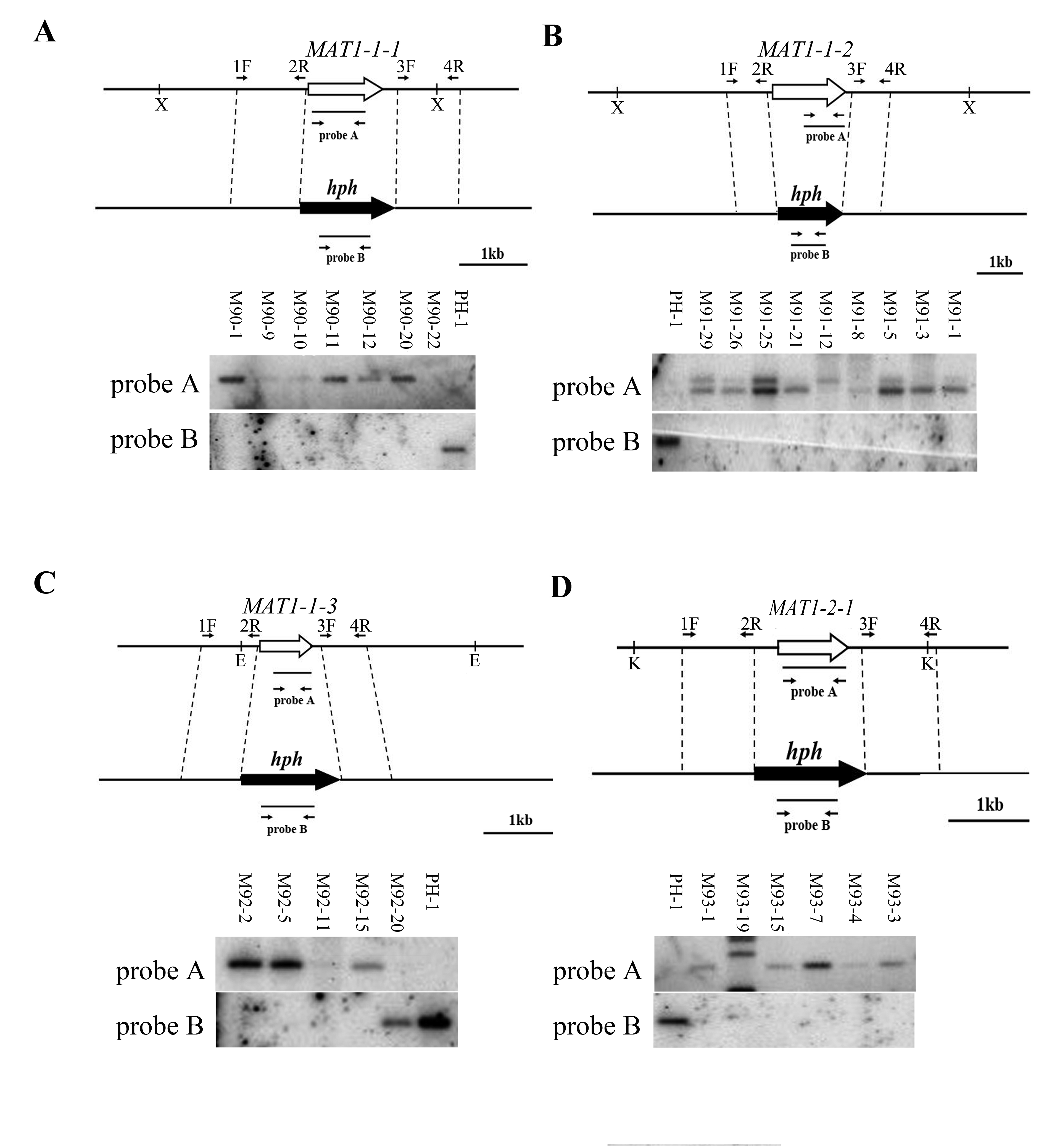

Supplement: Figure S2 — Generation of the gene replacement mutants of four MAT locus TF genes. A. TheMAT1-1-1 locus and gene replacement construct. TheMAT1-1-1 and hph genes are marked with empty and black arrows, respectively. 1F, 2R, 3F, and 4R are the primers used to amplify the flanking sequences. Lower panels are Southern blots of the wild type (PH-1) and putative mat1-1-1 mutants (M1, M2, and M3) hybridized with probe A (left) amplified with primers M15F/M16R and probe B (right) amplified with primers H852/H850. Panels B, C, and D were similar figures showing the gene replacement constructs and mutants of the MAT1-1-2, MAT1-1-3, and MAT1-2-1 genes, respectively. E, EcoRI; K, KpnI; X, XbaI (TIF) [file pone.0066980.s002.tif]

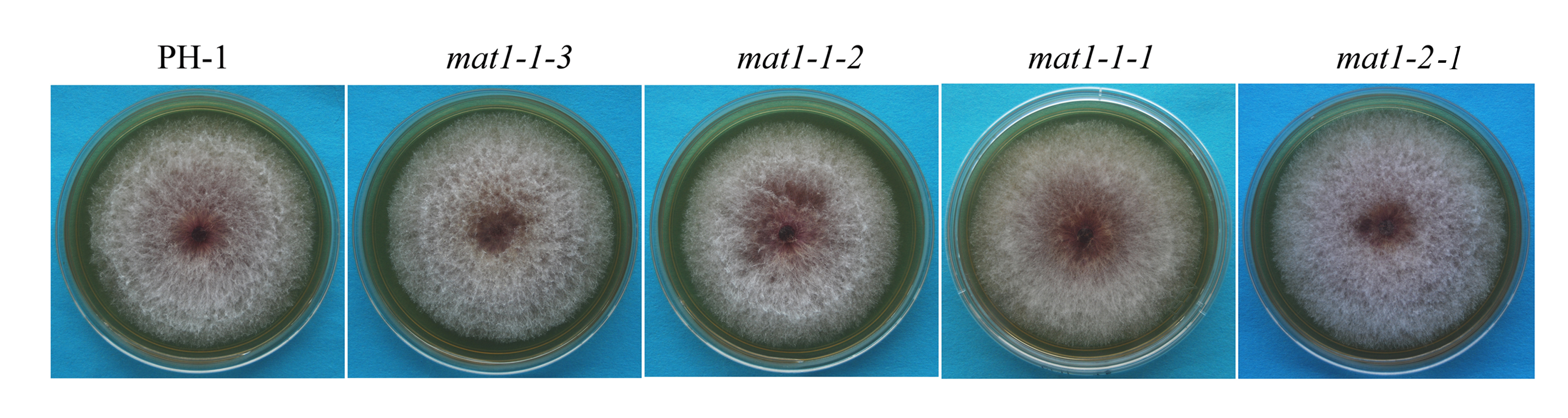

Supplement: Figure S3 — Three-day-old PDA cultures of the wild type and the mat1-1-3, mat1-1-2, mat1-1-1, and mat1-2-1 mutants. No differences in growth or colony morphology were observed between PH-1 and the mutants. (TIF) [file pone.0066980.s003.tif]

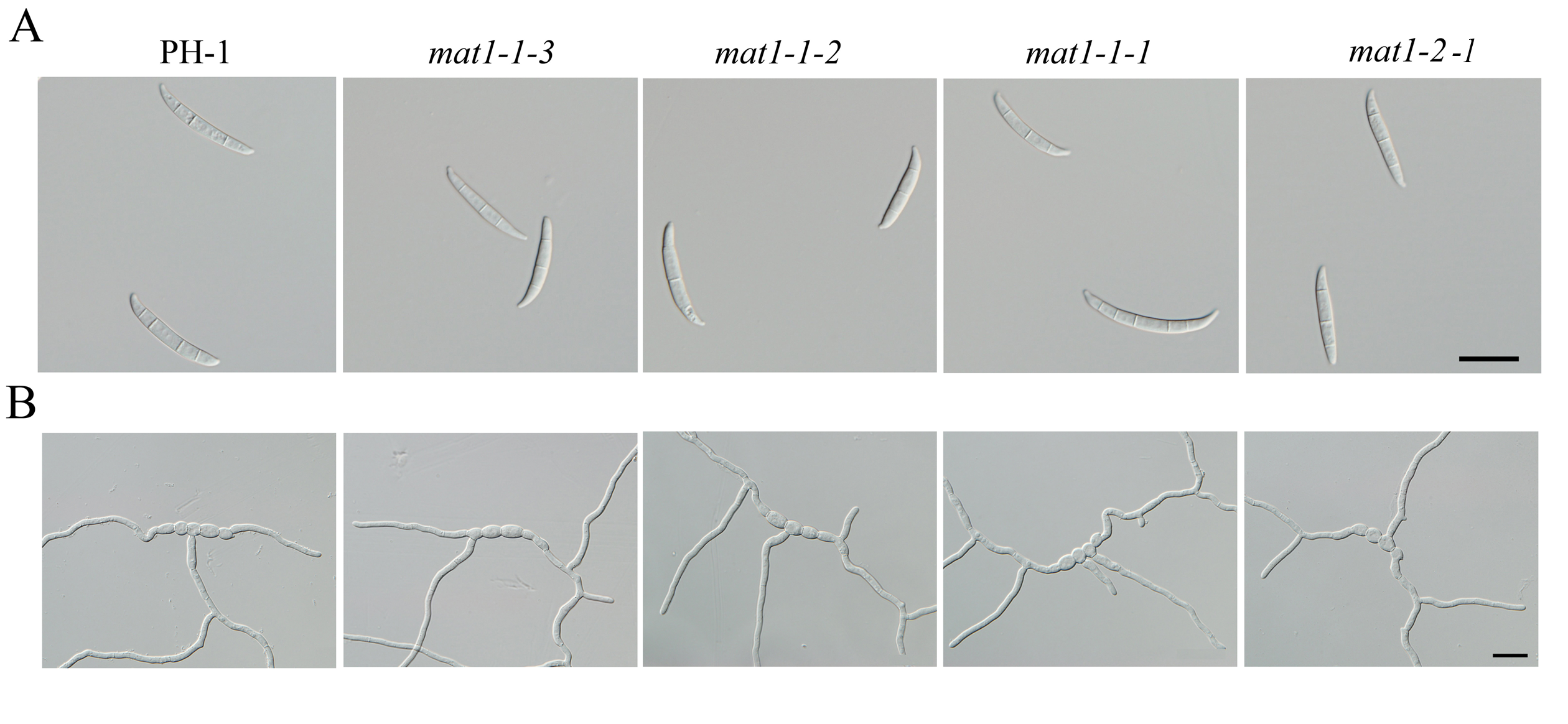

Supplement: Figure S4 — Conidia and 12 h germ tubes of the wild type (PH-1) and the mat1-1-3, mat1-1-2, mat1-1-1, and mat1-2-1 mutants. Bar = 20 µm. (TIF) [file pone.0066980.s004.tif]

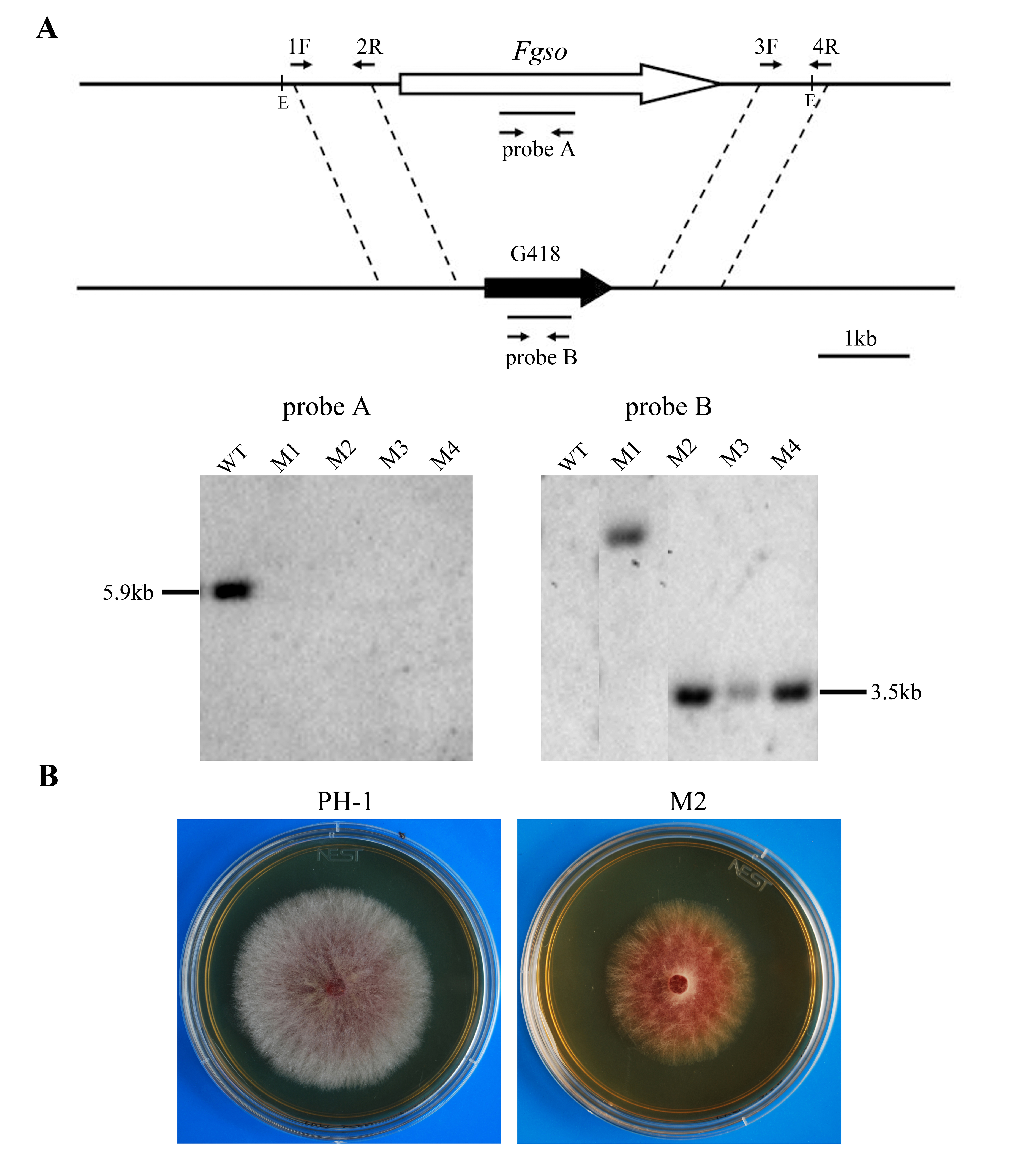

Supplement: Figure S5 — Generation of the Fgso deletion mutant. A. The FgSO gene replacement construct (upper panel) and verification of the Fgso deletion mutants by Southern blot analysis (lower panel). Genomic DNA samples were digested with EcoRV (E). WT, the wild type strain PH-1. M1-M4, putative Fgso mutants. B. Three-day-old PDA cultures of PH-1 and the Fgso deletion mutant. (TIF) [file pone.0066980.s005.tif]

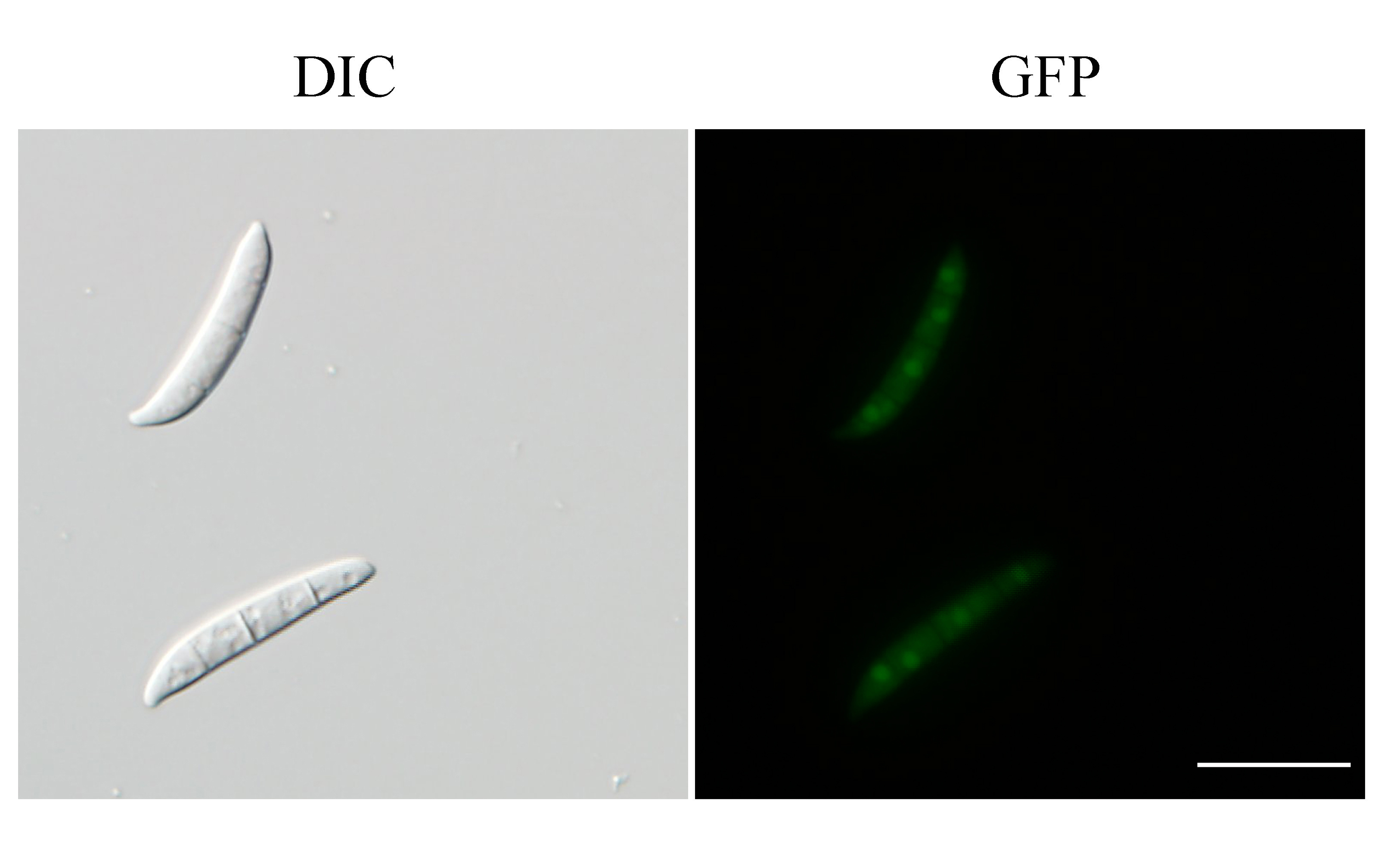

Supplement: Figure S6 — GFP signals in the conidia of PTrpC- MAT1-2-1 -GFP transformant. The localization of GFP signals in the nucleus and cytoplasm of conidia harvested from 5-day-old CMC cultures of the PTrpC-MAT1-2-1-GFP transformant. (TIF) [file pone.0066980.s006.tif]
